# Cardiovascular health and the modifiable burden of incident myocardial infarction: the Tromsø Study

**DOI:** 10.1186/s12889-015-1573-0

**Published:** 2015-03-06

**Authors:** Tom Wilsgaard, Laura R Loehr, Ellisiv B Mathiesen, Maja-Lisa Løchen, Kaare H Bønaa, Inger Njølstad, Gerardo Heiss

**Affiliations:** Department of Community Medicine, UiT The Arctic University of Norway, Tromsø, Norway; Department of Epidemiology, University of North Carolina at Chapel Hill, Chapel Hill, NC 27514 USA; Department of Clinical Medicine, UiT The Arctic University of Norway, Tromsø, Norway; Department of Cancer Research and Molecular Medicine, Norwegian University of Science and Technology, Trondheim, Norway

**Keywords:** Coronary heart disease, Cohort studies, Lifestyle, Longitudinal studies, Prevention

## Abstract

**Background:**

The American Heart Association has proposed an impact goal for the year 2020 to improve cardiovascular health by 20%. The objectives of the study were to assess the association between the proposed cardiovascular health metric score and incident myocardial infarction (MI) and to estimate the generalized impact fraction (GIF).

**Methods:**

The health metric score was derived from ideal levels of six cardiovascular risk factors from the population-based Tromsø Study of 22,121 residents of Tromsø, Norway aged 30 to 79 years, examined in 1994–95, 2001, and 2007–08. Incident events of MI were recorded from the date of enrollment in 1994–95 to the end of 2010. Adjudication of hospitalized and out-of hospital events was performed by an independent endpoints committee based on data from hospital and out-of hospital journals, autopsy records and death certificates. Cox proportional hazard regression was used to estimate hazard ratios (HR). GIF was calculated from age stratified analysis using a case-load weighted-sum method. Bootstrapping was used to estimate 95% simulation intervals.

**Results:**

A total of 1652 MIs accrued over an average of 14.7 person-years of follow-up. Few men (0.96%) and women (3.6%) had ideal levels in all 6 metrics. 64.7% (men) and 55.7% (women) had ideal levels in 2 or 3 metrics. The age-adjusted HR per point increase in health score was 0.65 (95% confidence interval: 0.61, 0.70) in men and 0.59 (0.54, 0.64) in women. A shift of 30% of subjects from low score levels ≤3 to scores ≥4 was estimated to prevent 13.7% (11.2, 16.2) of incident MI in men and 15.9% (12.1, 19.4) in women.

**Conclusions:**

The association between the health metric score and MI indicate that close to 15% of incident MIs could be prevented by attainable and realistic improvements in the health metrics.

**Electronic supplementary material:**

The online version of this article (doi:10.1186/s12889-015-1573-0) contains supplementary material, which is available to authorized users.

## Background

Trends in the population burden of cardiovascular disease (CVD) and associated lifestyle factors differ between regions of the world [1-4]. Studies of the temporal association of these patterns suggest that changes in lifestyle factors precede the change in CVD outcomes. Effective cardiovascular treatment also contributes to the decline in CVD mortality in Western Europe and USA [2]. A focus on a population’s cardiovascular health to prevent or postpone disabilities, years of life lost, and medical costs attributable to cardiovascular outcomes is thus given new impetus by the substantial trends observed in populations. For example, the American Heart Association (AHA) has in the ‘cardiovascular health’ construct recommended an impact goal for health promotion and disease reduction through 2020 and beyond [5]. The AHA defined ideal cardiovascular health by both ideal health behaviors (non-smoking, body mass index <25 kg/m^2^, physical activity at goal levels (≥150 min/week moderate intensity or ≥75 min/week vigorous intensity or ≥ 150 min/week moderate + vigorous), and pursuit of a diet consistent with current guideline recommendations) and ideal health factors (untreated total cholesterol <200 mg/dL, untreated blood pressure <120/<80 mmHg, and fasting blood glucose <100 mg/dL). The sum of these ideal health metrics was shown to be inversely associated with incident cardiovascular disease [6] although the impact of a population-level increase in these ideal health metrics on the potential prevention of cardiovascular disease has not been established. To our knowledge, no study has yet estimated the attributable fraction (AF) of ideal health metrics, i.e. the proportional reduction in disease given complete elimination of an exposure [7]. Moreover, given that complete elimination, as assumed in calculation of the AF, is not realistic for many exposures and that elimination of high risk factor levels from society is unlikely [8], a more realistic and meaningful estimation of the impact of risk factor reduction on disease incidence is the generalized impact fraction (GIF), also known as the potential impact fraction or the generalized AF [9,10]. It estimates the proportional reduction in disease incidence given a graded reduction in the prevalence of a risk factor or given a graded increase in the number of ideal health metrics. For common health behaviors, risk factors and diseases, the impact of a modest differences in population profiles may reveal substantial effects on disease incidence, even when risk factor-disease associations are relatively weak [11,12].

Our aim was to use data from the Tromsø Study in 1994–95, 2001, and 2008–09 to estimate the association between the number and type of cardiovascular health metrics and the risk of myocardial infarction (MI), and to quantify the population burden of MI that would be prevented at increasing numbers of cardiovascular disease health metrics in the population.

## Methods

### Study population

The population-based, prospective Tromsø Study consists of six surveys referred to as Tromsø 1–6, conducted in the municipality of Tromsø, Norway from 1974 to 2008 [13]. All men and women aged ≥25 years living in the municipality were invited to Tromsø 4 in 1994–95; Tromsø 4 is the baseline population for this study. For Tromsø 4, 27,158 subjects (72% of those invited) attended the study visit. The following participants were excluded from analyses: 1) younger than 30 years or older than 79 years of age (n = 3934), 2) did not consent to medical research (n = 166), 3) were pregnant (n = 160), 4) had missing values for cardiovascular disease risk factors (n = 132), 5) had prevalent MI (n = 613), or 6) had officially moved out of the municipality prior to their date of examination (n = 32). Thus, n = 22,121 men and women were included in the present analyses. Participants that were still under follow-up for MI and attended the later surveys in 2001 (Tromsø 5, n = 6455) and/or in 2007–08 (Tromsø 6, n = 8221) had their cardiovascular risk factor values updated at the date of their examination. The Tromsø Study was approved by the Data Inspectorate of Norway and the Regional Committee of Medical and Health Research Ethics, North Norway. Participation was voluntary and each subject gave written informed consent prior to participation.

### Measurements

Each survey used a standardized protocol of nearly identical methods including physical examination, blood sampling and administration of questionnaires. Blood pressure was measured with an automated device; three readings were recorded with one-minute intervals and the mean of the two final recordings were used in the analysis. Weight was measured to the nearest half kilogram (nearest decimal in Tromsø 6) with subjects wearing light clothing and no shoes. Non-fasting blood samples were analyzed by standard methods at the Department of Laboratory Medicine, University Hospital of North Norway. Smoking status was defined by self-report. In Tromsø 4, participants were asked the three questions “Do you smoke cigarettes/cigars/a pipe daily?”. In Tromsø 5 and 6, the question was “Do you smoke daily?”. A response of “Yes” to any of these questions indicated daily smoking. Leisure-time physical activity was defined from different questions in Tromsø 4 and 5 as compared to Tromsø 6 [14]. The number of minutes per week of light or hard activity was estimated and the ideal physical activity metric was defined as ≥150 minutes per week with light activity or ≥75 minutes per week with hard activity.

### Identification and validation of incident MI

Incident cases of MI among the participants were recorded from the date of enrollment in 1994–95 to the end of follow-up, December 31, 2010. Adjudication of hospitalized and out-of hospital events was performed by an independent endpoint committee based on records from hospital and out-of hospital settings, autopsy records and death certificates. The Norwegian national 11-digit identification number allowed linkage to national and local diagnosis registries. Cases of incident and prevalent MI were identified by linkage to the discharge diagnosis registry at the University Hospital of North Norway with search for ICD 8 codes 410–414, 427, 795–796 in the period 1969–1979, ICD 9 codes 410–414, 427.5, 798 and 799 in the period 1980–98, and thereafter ICD 10 codes I20–I25, I46, R96, R98 and R99. The University Hospital is the only hospital in the area serving the Tromsø population. Modified WHO MONICA/MORGAM criteria for MI were used and included clinical symptoms and signs, findings in electrocardiograms, values of cardiac biomarkers and autopsy reports when applicable. Linkage to the National Causes of Death Registry at Statistics Norway allowed identification of fatal incident cases of MI that occurred as out-of-hospital deaths, including deaths that occurred outside of Tromsø, as well as information on all-cause mortality. Information from the death certificates was used to collect relevant information of the event from additional sources such as autopsy reports and records from nursing homes, ambulance services and general practitioners. Dates of emigration were obtained from the Population Registry of Norway.

### Health metric score

The proposed Health Metric includes seven components [5]. However, its dietary component was not included in the present study because our cohort characterization lacked suitable questionnaires on dietary intake. Furthermore, because our blood assays were non-fasting, self-reported diabetes was used in place of fasting serum glucose. Our health metric score was thus defined as the number of ideal health levels for the following six cardiovascular disease risk factors: 1) BMI <25 kg/m^2^; 2) Total cholesterol <5.18 mmol/l (<200 mg/dl); 3) Systolic blood pressure <120 mmHg and diastolic blood pressure <80 mmHg; 4) Non-smokers; 5) Moderate physical activity ≥3 hours per week or vigorous physical activity ≥1 hours per week; and 6) Not diabetic.

### Statistical methods

All statistical analyses were performed using SAS software 9.3, SAS Institute Inc., Cary, NC, USA. Follow-up time extended from the day of study entry in 1994–95 to the date of first fatal or nonfatal MI event, participant censoring due to loss-to-follow up, death, or end of follow-up at December 31, 2010, whichever date was earliest. Cox proportional hazard regression was used to estimate hazard ratios (HRs) of incident MI using baseline cardiovascular health metric scores and updated repeated scores in 2001 and 2008–09 as time-dependent covariates. The cardiovascular health metric score was modeled both as an ordinal variable and by including each cardiovascular health metric score point as indicator variables compared to the reference level (0 metrics present). The GIF was introduced by Walter [15] in 1980 and described by Morgenstern et al. [11] in 1982, but has not been widely used and has not replaced or complemented the AF. In order to adjust for age, the GIF had to be estimated in age strata. The small number of MI events in the youngest 10 year age groups prevented an estimation of HR for MI for each health metric score level (0 to 6). Consequently, the score was categorized into four groups (≤1, 2, 3, ≥4). The overall GIF was estimated using the case-load weighted sum method [10] for the following three scenarios:

1. A 30% decrease in subjects with health metric levels ≤3, with movement of this 30% to the group with ideal health metric scores (≥4).
2. A 50% decrease in subjects with health metric levels ≤3, with movement of this 50% to the group with ideal health metric scores (≥4).
3. A 100% decrease in subjects with health metric levels ≤3, with movement of all 100% to the group with ideal health metric scores (≥4).

The latter scenario represents the AF. Bootstrapping was used to estimate 95% simulation intervals (SIs) for the GIF and AF. Bootstrapping was also used to assess possible GIF differences between men and women or between age groups. In supplemental models HRs and GIFs was calculated for each component of the health metric score.

The proportional hazard assumption was verified by visual inspection of log minus log survival curves and by tests of Schoenfeld residuals.

## Results

### Descriptive characteristics and HRs for individual health metrics

The average follow-up was 14.7 person-years. Mean age at baseline was 47.9 years in men and 48.9 years in women (Additional file 1). Mean BMI, systolic blood pressure, diastolic blood pressure (in men), physical inactivity, and diabetes increased between Tromsø 4 (1994–95) and Tromsø 6 (2007–08) for those still under follow-up, whereas total cholesterol, diastolic blood pressure (in women), and current smoking decreased (Additional file 1).

Subjects who attended follow-up examinations in 2001 or 2007–08 and did not experience a MI before the follow-up examination(s) had their cardiovascular health metrics updated. As shown in Table 1, the proportions of subject who had ideal levels of individual cardiovascular health metrics, at last updated value, were lowest in participants with incident MI. Blood pressure was the health metric with the smallest proportion within ideal limits (in men and women with MI: 5.7% and 4.9%; in men and women with-out MI: 12.6% and 27.7%, respectively). The combined proportions with ideal metrics in men and women were as follows: BMI, 38.5% and 51%; total cholesterol, 27.1% and 26.6%; blood pressure, 11.9% and 26.6%; Smoking, 67.7% and 68.4%; physical activity, 49.3% and 45.8%; and diabetes, 95.7% and 95.9%. Participants with ideal levels had a reduced hazard of MI for all metrics with age adjusted HRs ranging from 0.53 (non-diabetes) to 0.73 (ideal BMI) in men and 0.35 (ideal blood pressure) to 0.80 (ideal BMI) in women.

**Table 1:** **Distribution of ideal health metrics and hazard ratios for MI by sex**

	**Men (1056 MI cases in 10,537 participants)**			**Women (596 MI cases in 11,584 participants)**		
**Characteristic**	**% in MI cases**	**% in non- cases**	HR (95 % CI)*	**% in MI cases**	**% in non- cases**	HR (95 % CI)*
Body Mass Index < 25 kg/m^2^	31.0	39.4	0.73 (0.64, 0.83)	35.2	51.8	0.80 (0.68, 0.95)
Total cholesterol < 200 mg/dl	11.5	28.9	0.54 (0.44, 0.65)	7.0	27.7	0.57 (0.41, 0.78)
Ideal Blood pressure†	5.7	12.6	0.62 (0.48, 0.81)	4.9	29.2	0.35 (0.24, 0.52)
Non smoker	56.4	68.9	0.54 (0.48, 0.62)	59.1	68.9	0.40 (0.34, 0.47)
Physical activity‡	49.1	49.3	0.78 (0.69, 0.88)	35.2	46.4	0.72 (0.61, 0.86)
Non - Diabetes	92.5	96.1	0.53 (0.42, 0.67)	89.3	96.2	0.44 (0.34, 0.58)

MI, myocardial infarction; HR, hazard ratio; CI confidence interval.

*Age adjusted Cox proportional hazard regression models. The health metrics were updated for those who attended the follow-up examinations in 2001 or 2007–08 and did not experience an event prior to the follow-up examination.

†Systolic blood pressure < 120 mmHg and diastolic blood pressure < 80 mmHg.

‡Moderate physical activity ≥ 3 hours per week or vigorous physical activity ≥ 1 hour per week.

The Tromsø Study 1994-2008.

### HRs according to health metric score

As shown in Table 2, 101 men (0.96%) and 417 women (3.6%) had ideal levels in all 6 cardiovascular health metrics. The majority of participants had 2 or 3 ideal levels, 64.7% (men) and 55.7% (women). The hazard for MI decreased significantly by increasing health metric score in both men and women, age adjusted HR per score point increase 0.65 (95% confidence interval (CI): 0.61, 0.70) in men and 0.59 (0.54, 0.64) in women.

**Table 2:** Hazard ratios for MI according to health metric score* by sex

**Health metrics**	**Men, n = 10,537**				**Women, n = 11,584**			
**score**	**%†**	**No. of MI**	**IR‡**	**HR (95 % CI)§**	**%†**	**No. of MI**	**IR‡**	**HR (95 % CI)§**
= 0	0.2	6	2653	Ref	0.3	6	928	Ref
= 1	7.9	182	1715	0.63 (0.28, 1.43)	6.3	100	617	0.65 (0.28, 1.47)
= 2	28.9	343	821	0.30 (0.14, 0.68)	25.8	251	312	0.33 (0.15, 0.73)
= 3	35.8	382	671	0.25 (0.11, 0.56)	30.9	185	224	0.23 (0.10, 0.53)
= 4	19.9	130	460	0.17 (0.07, 0.39)	22.0	52	126	0.13 (0.06, 0.30)
= 5	6.4	12	170	0.06 (0.02, 0.17)	11.0	2	18	0.02 (0.00, 0.09)
= 6	1.0	1	109	0.04 (0.00, 0.34)	3.6	0	0	NA
Overall||	100	1056	633	0.65 (0.61, 0.70)	100	596	201	0.59 (0.54, 0.64)

MI, myocardial infarction; IR, incidence rate, HR, hazard ratio; CI confidence interval.

*Health Metric Score is defined as the number of ideal health levels for six cardiovascular disease risk factors.

1) Body Mass Index < 25 kg/m^2^.

2) Total cholesterol < 5.18 mmol/l (<200 mg/dl).

3) Systolic blood pressure < 120 mmHg and diastolic blood pressure < 80 mmHg.

4) Non-smokers.

5) Moderate physical activity ≥ 3 hours per week or vigorous physical activity ≥ 1 hours per week.

6) Non – Diabetes.

†Percent of participants.

‡Age adjusted incidence rate per 100,000 person-years from poisson regression.

§Age adjusted Cox proportional hazard regression models with updated values of health metrics score during follow-up using participants with score ≤ 1 as the reference level.

||HR per unit increase in health metric score.

The Tromsø Study 1994–2008.

MI incidence, although lower in women, increased exponentially (base 2) per 10-year age group (Additional file 2). The health metric score distribution varied slightly between the age groups. However, in women, the HR for MI weakened by age for most health metric score groups using participants with a score ≥4 as the reference group. In men, the trend of weaker HRs by age was not as clear, especially because the 50–59 years age group were observed with the highest HRs compared to the reference group shown as follows: score group equal to 0 or 1, HR 6.82 (95% CI: 4.10, 11.4); score group equal to 2, HR 3.26 (2.01, 5.30); score group equal to 3, HR 2.77 (1.71, 4.50). In women, the corresponding HRs in the 50–59 years age groups were: 8.35 (4.21, 16.6), 3.19 (1.65, 6.19), and 2.22 (1.12, 4.38).

### GIF according to decrease in unfavorable health metric score levels

As shown in Table 3, the estimated proportion of prevented incident MI (GIF) varied by different scenarios of population increase in health metric score and by age. In scenario 1, a 30% decrease in subjects in each score level ≤3 to the ideal score group (scores ≥4) could prevent 13.7% (95% SI: 11.2, 16.2) of incident MI in men and 15.9% (12.1, 19.4) in women. These GIFs translate to 105 prevented MIs per 100,000 person-years in men and 59 in women. A 50% decrease in subjects in each score level ≤3 to the ideal score group (scores ≥4) could prevent 22.9% (18.6, 27.0) of incident MI in men (175 prevented MIs) and 26.5% (20.0, 32.3) in women (99 prevented MIs). Finally, scenario 3 represents the AF and assumes a complete elimination of scores lower than 4, and suggests that 45.8% (37.3, 53.9) of incident MI could be prevented in men and 53.1% (40.3, 64.6) in women. The observed GIF difference between men and women was not significant, p = 0.61. However, the GIFs in those aged 60–79 years were significantly lower than those younger than 60 years of age, p = 0.024 in women and p = 0.002 in men.

**Table 3:** Generalized Impact Fraction of decrease in unfavorable levels of Health Metric Score* by age and sex

	**Scenario 1†**		**Scenario 2‡**		**Scenario 3§**	
**Baseline age, years**	**GIF (95% SI)**	**Prev, no||**	**GIF (95% SI)**	**Prev, no||**	**GIF (95% SI)**	**Prev, no||**
Men						
30 – 39	15.8 (9.3, 22.0)	32	26.4 (15.4, 36.7)	53	52.8 (30.9, 73.4)	106
40 – 49	15.3 (9.8, 20.4)	64	25.5 (16.3, 34.0)	106	51.1 (32.6, 67.9)	213
50 – 59	19.8 (15.4, 23.9)	185	33.0 (25.7, 39.9)	308	66.0 (51.4, 79.7)	616
60 – 69	8.3 (2.4, 13.7)	153	13.9 (4.1, 22.8)	256	27.8 (8.2, 45.6)	512
70 – 79	11.4 (5.3, 16.8)	406	18.9 (8.9, 28.0)	673	37.9 (17.8, 56.1)	1350
Overall**	13.7 (11.2, 16.2)	105	22.9 (18.6, 27.0)	175	45.8 (37.3, 53.9)	351
Women						
30 – 39	NA	NA	NA	NA	NA	NA
40 – 49	25.5 (20.8, 28.9)	30	42.5 (34.6, 48.2)	49	85.1 (69.2, 96.3)	99
50 – 59	19.4 (13.2, 25.0)	76	32.4 (22.0, 41.7)	128	64.8 (44.0, 83.5)	256
60 – 69	11.3 (3.3, 18.4)	105	18.8 (5.5, 30.6)	175	37.5 (11.0, 61.3)	350
70 – 79	15.6 (9.0, 21.5)	281	26.0 (15.0, 35.9)	468	52.0 (30.1, 71.7)	936
Overall**	15.9 (12.1, 19.4)	59	26.5 (20.1, 32.3)	99	53.1 (40.3, 64.6)	197

GIF, generalized impact fraction in percent; SI, 2.5 % to 97.5% simulation interval from 10,000 bootstrapped data sets.

*Health Metric Score is defined as the number of ideal health levels for six cardiovascular disease risk factors.

1) Body Mass Index < 25 kg/m^2^.

2) Total cholesterol < 5.18 mmol/l (<200 mg/dl).

3) Systolic blood pressure < 120 mmHg and diastolic blood pressure < 80 mmHg.

4) Non-smokers.

5) Moderate physical activity ≥ 3 hours per week or vigorous physical activity ≥ 1 hours per week.

6) Non – Diabetes.

†30% decrease in subjects with levels ≤3, with movement of this 30% to the group with ideal scores (≥4).

‡50% decrease in subjects with levels ≤3, with movement of this 50% to the group with ideal scores (≥4).

§100% decrease in subjects with levels ≤3, with movement of this 100% to the group with ideal scores (≥4).

||The preventable number of MIs per 100,000 person-years.

**The overall GIF using the case-load weighted sum method.

The Tromsø Study 1994–2008.

Although the age specific GIFs are higher in the younger age groups the preventable numbers of MIs are higher in the older age groups (Table 3), as would be expected from the differences in risk factor levels and the exponential increase in MI incidence over age (Additional file 2). Using scenario 1, 406 MIs per 100,000 person-years could be prevented in men age 70–79 years at baseline as compared to 64 prevented MIs in the 40 – 49 year age group. The corresponding figures in women are 281 vs 30 prevented MIs.

### GIF according to individual health metrics

Additional files 3, 4, 5, 6, 7 and 8 show age and sex specific estimated proportions of prevented incident MI (the generalized impact fraction, GIF) for each health metric and the overall GIF for each health metric are summarized in Table 4. The highest GIFs were observed for blood pressure and the lowest for diabetes. A 30% decrease in subjects not in ideal blood pressure levels suggest a prevention of 10.4% (95% SI: 4.6, 15.3) of incident MI in men and 15.1% (8.1, 20.7) in women, whereas the corresponding figures were 2.2% (1.3, 3.1) and 2.8% (1.7, 4.0) for diabetes. Women were observed to have higher GIFs for blood pressure, smoking, physical activity, and diabetes than did men, while BMI and total cholesterol had higher GIFs in men.

**Table 4:** Overall Generalized Impact Fraction* of reduction in unfavorable levels of cardiovascular health metrics by sex

	**Scenario 1‡**		**Scenario 2§**		**Scenario 3||**	
**Cardiovascular health metrics†**	**GIF (95% SI)**	Prev, no**	**GIF (95% SI)**	Prev, no**	**GIF (95% SI)**	Prev, no**
Men						
Body mass index	6.2 (4.0, 8.3)	48	10.4 (6.7, 13.8)	80	20.7 (13.5, 27.7)	159
Cholesterol	9.9 (6.6, 13.1)	76	16.4 (11.0, 21.8)	126	32.9 (22.0, 43.6)	252
Blood pressure	10.4 (4.6, 15.3)	80	17.3 (7.7, 25.5)	133	34.6 (15.5, 51.1)	265
Smoking	5.5 (4.3, 6.8)	43	9.3 (7.2, 11.4)	71	18.5 (14.3, 22.8)	142
Physical activity	3.9 (2.0, 5.7)	30	6.6 (3.4, 9.5)	50	13.1 (6.8, 18.9)	100
Diabetes	2.2 (1.3, 3.1)	15	3.6 (2.2, 5.2)	26	7.2 (4.4, 10.3)	51
Women						
Body mass index	3.2 (0.4, 6.1)	12	5.4 (0.6, 10.1)	20	10.8 (1.2, 20.2)	40
Cholesterol	6.7 (−1.1, 13.7)	25	11.2 (−1.8, 22.8)	42	22.3 (−3.5, 45.5)	84
Blood pressure	15.1 (8.1, 20.7)	57	25.2 (13.5, 34.5)	95	50.4 (27.0, 69.0)	189
Smoking	7.1 (5.6, 8.7)	27	11.9 (9.3, 14.5)	44	23.8 (18.6, 29.1)	88
Physical activity	5.4 (2.4, 8.1)	20	9.0 (4.0, 13.5)	34	17.8 (8.1, 26.9)	67
Diabetes	2.8 (1.7, 4.0)	10	4.7 (2.8, 6.6)	17	9.3 (5.7, 13.2)	35

GIF, Generalized Impact Fraction in percent; SI, 2.5 % to 97.5% Simulation Interval from 10,000 bootstrapped data sets.

*The overall GIF using the case-load weighted sum method.

†Ideal levels of the health metrics.

Body Mass Index < 25 kg/m^2^.

Total cholesterol < 5.18 mmol/l (<200 mg/dl).

Systolic blood pressure < 120 mmHg and diastolic blood pressure < 80 mmHg.

Non-smokers.

Moderate physical activity ≥ 3 hours per week or vigorous physical activity ≥ 1 hours per week.

Non – Diabetes.

‡30% decrease in subjects not in ideal levels, with movement of this 30% to ideal levels.

§50% decrease in subjects not in ideal levels, with movement of this 50% to ideal levels.

||100% elimination of subjects not in ideal levels, with movement of this 100% to ideal levels.

**The preventable number of MI per 100,000 person-years.

The Tromsø Study 1994–2008.

## Discussion

We found a significant linear association between the number of ideal health metrics and subsequent incident MI in men and women. The observed hazard ratios and the incidence of age specific MI were used to estimate the generalized impact fraction. Our results can be interpreted as a hypothetical shift of 30% of subjects from the group with low health metrics scores (≤3) to the group with ideal health metric scores (≥4) and was estimated to prevent 13.7% (95% SI: 11.2, 16.2) of incident MIs in men and 15.9% (12.1, 19.4) in women.

The burden of CVD in Norway and the majority of Western societies in terms of life-years lost, diminished quality of life, and direct and indirect medical costs is high [16,17]. The average costs of acute fatal and non-fatal MI in Norway was estimated to 43,425 NOK (7200 USD) and 114,932 NOK (19,100 USD), respectively, in 2005 [17]. Statistics Norway reported that 4852 persons died of ischemic heart disease in Norway in 2012. If we assume a 20% case fatality [18], 19,408 persons would have experienced a non-fatal ischemic heart attack that year. By extrapolation results for scenario 1, a shift of 30% of subjects from low health metrics score levels <4 to scores ≥4, a GIF of 15% would have resulted in 3639 fewer fatal and non-fatal heart attacks in Norway. In terms of medical costs potentially averted, 3639 heart attacks translate to more than 366 million NOK or approximately 52 million USD per year, without considering stroke, atrial fibrillation, heart failure, angina pectoris and type 2 diabetes.

The 7 health metrics considered by the AHA Strategic Planning Task Force [5] are at unfavorable levels at present in Norway [19] and in the US [20]. However, such trends are dynamic [21,22] and complex in their response to social norms, public education, and market influences [23]. Understanding their potential impact on the health of populations is therefore an important focus of attention for public health and policy, to complement established but insufficient medical models of risk factor reduction [24-26].

We are not aware of any reported estimates of the GIF of the health metrics score in relation to cardiovascular disease. Some studies considered fewer health metrics and assessed the burden of cardiovascular disease attributed to an accumulation of elevated risk factors, or assessed the preventive effect associated with a combination of favorable levels of risk factors [27-30]. Common to these studies is their empirical support for the prognostic importance of the cardiovascular health metric construct [31].

The association between the health metric score and CVD using all 7 proposed health metrics was assessed by Folsom et al. [6] showing a gradient of decreasing incidence of CVD for increasing numbers of ideal health metrics, in line with our results. Very few members of the ARIC cohort had all 7 cardiovascular components in the ideal range (0.1%), similar to our results indicating that very few residents of Tromsø have ideal cardiovascular health, as only 2.3% had a maximum score of 6. Although Folsom et al. did not present the GIF of the health metrics in relation to CVD, their results agree with ours and, thus, strengthen the generalizability of our results.

In the absence of empirical information it is difficult to determine feasible goals for population-wide reduction in unfavorable levels in several cardiovascular health metrics. We have reported scenarios of 30%, 50%, and 100% decrease in subjects in each score level < 4 to the ideal score group (scores ≥ 4). If the AHA goal for the year 2020 is equivalent to a GIF of 20%, reductions of a magnitude between our first two scenarios may have to be met. This goal may be too optimistic, although the recent and rapid population-wide changes in lifestyle related health metrics speak to the dynamic nature of these processes. A 30% reduction could be attainable but may require a downturn of the observed “obesity epidemic”. A study by Huffman et al. concludes that the AHA goal for 2020 will not be reached if current trends in the individual cardiovascular health metrics continue [32]. These authors discuss targets for interventions and emphasize that a “reversal of body weight trends will have substantial benefits across the spectrum of cardiovascular health”.

The strengths of our study include the population based, prospective design with high participation proportion (72% in 1994–95), the high sensitivity derived from the use of the Norwegian unique personal identity number to search registries, the use of adjudicated first-ever MI of the only local hospital, the inclusion of both hospitalized and out-of-hospital events, and the standardized and repeated update of CVD risk factors. Limitations of these analyses include the use of stratified analyses for the estimation of the GIFs, with some loss of precision at younger ages, which required collapsing health metric score levels 4, 5, and 6. The stratification made it possible to adjust for age as a confounder. Other non-stratified types of covariate adjustment such as modelling cannot be used in assessing the GIF. However, this limitation may be limited considering the fact the most traditional risk factors for CVD are included in the health metric score. A measurement bias could have influenced our results by a slight attenuation of HRs, especially for the self-reported variables (smoking, physical activity, and diabetes). Lastly, the Tromsø population is a relatively homogenous, middle-aged Caucasian population, which may constrain the generalizability of our estimates to other ethnic groups.

## Conclusions

A graded association was shown between the cardiovascular health metric score and incident MI in this population-based longitudinal study from Norway. The GIFs indicate that 13.7% of the MIs in men and 15.9% of those in women may be prevented if 30% of those with unfavorable levels of health metrics score levels (≤3) shift to ideal levels (≥4). A 50% transfer from low levels to ideal levels (≥4) is estimated to prevent 22.9% of incident MI in men, and 26.5% in women.

## Additional files

Additional file 1: Table S1. Characteristics of risk factors by sex and survey. The Tromsø Study 1994-2008. Additional file 2: Table S2. Incidence rates and hazard ratios with 95 % confidence intervals for MI according to Health Metric Score* levels by age and sex. The Tromsø Study 1994-2008. Additional file 3: Table S3. Generalized Impact Fraction of reduction in BMI by age and sex. The Tromsø Study 1994-2008. Additional file 4: Table S4. Generalized Impact Fraction of reduction in total cholesterol by age and sex. The Tromsø Study 1994-2008. Additional file 5: Table S5. Generalized Impact Fraction of reduction in blood pressure by age and sex. The Tromsø Study 1994-2008. Additional file 6: Table S6. Generalized Impact Fraction of reduction in smoking by age and sex. The Tromsø Study 1994-2008. Additional file 7: Table S7. Generalized Impact Fraction of a decrease in unfavorable physical activity levels by age and sex. The Tromsø Study 1994-2008. Additional file 8: Table S8. Generalized Impact Fraction of reduction in diabetes by age and sex. The Tromsø Study 1994-2008.
