# Supplementary material for: Cardiovascular health and the modifiable burden of incident myocardial infarction: the Tromsø Study
Source: BMC Public Health. 2015 Mar 6;15:221. doi: 10.1186/s12889-015-1573-0 (PMC4355366; doi:10.1186/s12889-015-1573-0)
Supplement: Additional file 1: Table S1. — Characteristics of risk factors by sex and survey. The Tromsø Study 1994-2008. [file 12889_2015_1573_MOESM1_ESM.docx]

Supplemental Table 1. Characteristics of risk factors by sex and survey. The Tromsø Study 1994-2008.

|  | Men | | |  | Women | | |
| --- | --- | --- | --- | --- | --- | --- | --- |
| Characteristic* | Tromsø 4 in 1994-95, n=10,537 | Tromsø 5 in 2001, n=2,659 | Tromsø 6 in 2007-08, n=3,736 |  | Tromsø 4 in 1994-95, n=10,537 | Tromsø 5 in 2001, n=2,659 | Tromsø 6 in 2007-08, n=3,736 |
| Age, years | 47.9 (12.4) | 62.3 (11.4) | 61.6 (9.6) |  | 48.9 (13.2) | 62.4 (11.2) | 62.2 (10.3) |
| Body Mass Index, kg/m^2^ | 25.7 (3.3) | 26.8 (3.6) | 27.2 (3.6) |  | 24.9 (4.3) | 26.8 (4.7) | 26.6 (4.6) |
| Total cholesterol, mmol/l | 6.1 (1.2) | 6.1 (1.1) | 5.6 (1.0) |  | 6.2 (1.4) | 6.4 (1.2) | 5.9 (1.1) |
| Systolic blood pressure, mmHg | 137.6 (17) | 141.6 (20) | 140.9 (21) |  | 132.9 (22) | 140.4 (23) | 139.1 (25) |
| Diastolic blood pressure, mmHg | 81.0 (11.6) | 82.5 (11.7) | 82.1 (10.2) |  | 77.3 (12.5) | 80.1 (12.7) | 75.9 (10.2) |
| Current smokers, % | 38.0 | 27.6 | 17.8 |  | 37.3 | 26.9 | 20.1 |
| Physical inactivity, % | 44.9 | 40.2 | 59.8 |  | 51.6 | 48.0 | 57.0 |
| Diabetes, % | 1.9 | 4.6 | 7.1 |  | 2.0 | 4.2 | 5.9 |

*Values are mean (standard deviation) or percent.
