# Supplementary material for: Cardiovascular health and the modifiable burden of incident myocardial infarction: the Tromsø Study
Source: BMC Public Health. 2015 Mar 6;15:221. doi: 10.1186/s12889-015-1573-0 (PMC4355366; doi:10.1186/s12889-015-1573-0)
Supplement: Additional file 2: Table S2. — Incidence rates and hazard ratios with 95 % confidence intervals for MI according to Health Metric Score* levels by age and sex. The Tromsø Study 1994-2008. [file 12889_2015_1573_MOESM2_ESM.docx]

Supplemental Table 2. Incidence rates and hazard ratios with 95 % confidence intervals for MI according to Health Metric Score* levels by age and sex. The Tromsø Study 1994-2008.

|  |  |  |  | Health Metric Score | | | | | | | |
| --- | --- | --- | --- | --- | --- | --- | --- | --- | --- | --- | --- |
|  |  |  |  | = 3 | |  | = 2 | |  | = 1 or 0 | |
| Baseline  age, years | No. of  participants | No. of  MI | IR† | %‡ | HR  (95% CI)§ |  | %‡ | HR  (95% CI)§ |  | %‡ | HR  (95% CI)§ |
| Men |  |  |  |  |  |  |  |  |  |  |  |
| 30 – 39 | 3152 | 85 | 201 | 33.1 | 1.45 (0.74, 2.84) |  | 24.4 | 2.76 (1.47, 5.19) |  | 6.6 | 9.16 (4.78, 17.5) |
| 40 – 49 | 3222 | 190 | 417 | 34.1 | 1.92 (1.20, 3.05) |  | 31.7 | 2.23 (1.41, 3.55) |  | 8.4 | 5.06 (3.07, 8.34) |
| 50 – 59 | 2096 | 261 | 934 | 38.4 | 2.77 (1.71, 4.50) |  | 30.7 | 3.26 (2.01, 5.30) |  | 9.8 | 6.82 (4.10, 11.4) |
| 60 – 69 | 1287 | 277 | 1842 | 39.4 | 1.18 (0.84, 1.67) |  | 30.2 | 1.42 (0.99, 2.02) |  | 8.1 | 3.31 (2.18, 5.01) |
| 70 – 79 | 780 | 243 | 3563 | 41.5 | 1.58 (1.08, 2.30) |  | 28.3 | 1.92 (1.29, 2.85) |  | 8.2 | 2.34 (1.41, 3.91) |
| Overall | 10,537 | 1056 | 767 | 35.4 | 1.70 (1.40, 2.06) |  | 30.0 | 2.08 (1.71, 2.53) |  | 8.5 | 4.40 (3.54, 5.47) |
| Women |  |  |  |  |  |  |  |  |  |  |  |
| 30 – 39 | 3407 | 6 | 13 | 25.2 | NA |  | 12.9 | NA |  | 3.3 | NA |
| 40 – 49 | 3332 | 56 | 116 | 30.7 | 4.29 (1.38, 13.3) |  | 22.4 | 8.86 (2.98, 26.3) |  | 6.6 | 45.2 (15.6, 131) |
| 50 – 59 | 2170 | 120 | 394 | 34.0 | 2.22 (1.12, 4.38) |  | 32.8 | 3.19 (1.65, 6.19) |  | 9.6 | 8.35 (4.21, 16.6) |
| 60 – 69 | 1548 | 191 | 933 | 37.3 | 1.40 (0.85, 2.28) |  | 39.6 | 1.84 (1.14, 2.96) |  | 8.4 | 2.45 (1.36, 4.43) |
| 70 – 79 | 1127 | 223 | 1799 | 34.4 | 1.87 (1.12, 3.14) |  | 42.1 | 2.40 (1.46, 3.96) |  | 8.4 | 3.30 (1.81, 6.02) |
| Overall | 11,584 | 596 | 376 | 30.4 | 2.25 (1.66, 3.06) |  | 27.1 | 3.13 (2.32, 4.22) |  | 7.2 | 6.34 (4.56, 8.83) |

MI, myocardial infarction; IR, incidence rate, HR, hazard ratio; CI confidence interval.

* Health Metric Score is defined as the number of ideal health levels for six cardiovascular disease risk factors

1. Body Mass Index < 25 kg/m^2^.
2. Total cholesterol < 5.18 mmol/l (< 200 mg/dl).
3. Systolic blood pressure < 120 mmHg and diastolic blood pressure < 80 mmHg.
4. Non-smokers.
5. Moderate physical activity ≥ 3 hours per week or vigorous physical activity ≥ 1 hours per week.
6. Non – Diabetes.

†Incidence rate per 100,000 person-years.

‡Percent participants within age group.

§Reference level ideal Health Metric Score = 4, 5 or 6. The overall HRs are adjusted for age.
