# Supplementary material for: Cardiovascular health and the modifiable burden of incident myocardial infarction: the Tromsø Study
Source: BMC Public Health. 2015 Mar 6;15:221. doi: 10.1186/s12889-015-1573-0 (PMC4355366; doi:10.1186/s12889-015-1573-0)
Supplement: Additional file 3: Table S3. — Generalized Impact Fraction of reduction in BMI by age and sex. The Tromsø Study 1994-2008. [file 12889_2015_1573_MOESM3_ESM.docx]

Supplemental Table 3. Generalized Impact Fraction of reduction in BMI by age and sex. The Tromsø Study 1994-2008.

|  | Scenario 1* | | Scenario 2† | | Scenario 3‡ | |
| --- | --- | --- | --- | --- | --- | --- |
| Baseline age, years | GIF (95% SI) | Prev, no§ | GIF (95% SI) | Prev, no§ | GIF (95% SI) | Prev, no§ |
| Men |  |  |  |  |  |  |
| 30 – 39 | 10.6 (4.4, 16.7) | 21 | 17.6 (7.3, 27.8) | 35 | 35.3 (14.6, 55.6) | 71 |
| 40 – 49 | 9.8 (4.9, 14.5) | 41 | 16.3 (8.2, 24.1) | 68 | 32.6 (16.3, 48.2) | 136 |
| 50 – 59 | 8.7 (3.8, 13.3) | 81 | 14.6 (6.4, 22.2) | 136 | 29.1 (12.7, 44.4) | 272 |
| 60 – 69 | 3.1 (-1.3, 7.4) | 57 | 5.2 (-2.2, 12.4) | 96 | 10.3 (-4.4, 24.8) | 190 |
| 70 – 79 | 2.8 (-1.4, 7.0) | 100 | 4.6 (-2.4, 11.7) | 164 | 9.3 (-4.8, 23.5) | 331 |
| Overall\|\| | 6.2 (4.0, 8.3) | 48 | 10.4 (6.7, 13.8) | 80 | 20.7 (13.5, 27.7) | 159 |
| Women |  |  |  |  |  |  |
| 30 – 39 | 9.4 (-6.9, 23.6) | 1 | 15.7 (-11.5, 39.4) | 2 | 31.4 (-23.0, 78.7) | 4 |
| 40 – 49 | 14.4 (7.9, 20.5) | 17 | 24.1 (13.1, 34.2) | 28 | 48.1 (26.3, 68.3) | 56 |
| 50 – 59 | 4.5 (-2.0, 10.8) | 18 | 7.4 (-3.4, 17.9) | 29 | 14.9 (-6.8, 35.9) | 59 |
| 60 – 69 | 1.2 (-4.2, 6.5) | 11 | 2.0 (-7.0, 10.8) | 19 | 4.0 (-13.9, 21.6) | 37 |
| 70 – 79 | 1.3 (-3.5, 6.2) | 23 | 2.2 (-5.8, 10.3) | 40 | 4.4 (-11.7, 20.7) | 79 |
| Overall\|\| | 3.2 (0.4, 6.1) | 12 | 5.4 (0.6, 10.1) | 20 | 10.8 (1.2, 20.2) | 40 |

GIF, Generalized Impact Fraction in percent; SI, 2.5 % to 97.5% Simulation Interval from 10,000 bootstrapped data sets.

*30% reduction in BMI ≥ 25 kg/m­­^2^ to BMI < 25 kg/m­­^2^.

†50% reduction in BMI ≥ 25 kg/m­­^2^ to BMI < 25 kg/m­­^2^.

‡100% reduction in BMI ≥ 25 kg/m­­^2^ to BMI < 25 kg/m­­^2^.

§The preventable number of MI per 100,000 person-years.

||The overall GIF using the case-load weighted sum method.
