# Supplementary material for: Cardiovascular health and the modifiable burden of incident myocardial infarction: the Tromsø Study
Source: BMC Public Health. 2015 Mar 6;15:221. doi: 10.1186/s12889-015-1573-0 (PMC4355366; doi:10.1186/s12889-015-1573-0)
Supplement: Additional file 4: Table S4. — Generalized Impact Fraction of reduction in total cholesterol by age and sex. The Tromsø Study 1994-2008. [file 12889_2015_1573_MOESM4_ESM.docx]

Supplemental Table 4. Generalized Impact Fraction of reduction in total cholesterol by age and sex. The Tromsø Study 1994-2008.

|  | Scenario 1* | | Scenario 2† | | Scenario 3‡ | |
| --- | --- | --- | --- | --- | --- | --- |
| Baseline age, years | GIF (95% SI) | Prev, no§ | GIF (95% SI) | Prev, no§ | GIF (95% SI) | Prev, no§ |
| Men |  |  |  |  |  |  |
| 30 – 39 | 19.1 (12.5, 24.9) | 38 | 31.8 (20.9, 41.6) | 64 | 63.7 (41.8, 83.1) | 128 |
| 40 – 49 | 16.0 (9.7, 21.7) | 67 | 26.7 (16.2, 36.2) | 111 | 53.3 (32.5, 72.5) | 222 |
| 50 – 59 | 14.1 (8.3, 19.6) | 132 | 23.5 (13.8, 32.6) | 220 | 46.9 (27.6, 65.2) | 438 |
| 60 – 69 | 5.6 (-1.1, 12.1) | 103 | 9.3 (-1.9, 20.2) | 171 | 18.7 (-3.7, 40.4) | 345 |
| 70 – 79 | 2.1 (-6.7, 10.2) | 75 | 3.6 (-11.1, 17.0) | 128 | 7.2 (-22.2, 34.0) | 257 |
| Overall\|\| | 9.9 (6.6, 13.1) | 76 | 16.4 (11.0, 21.8) | 126 | 32.9 (22.0, 43.6) | 252 |
| Women |  |  |  |  |  |  |
| 30 – 39 | 8.8 (-18.5, 22.4) | 1 | 14.7 (-30.8, 37.3) | 2 | 29.5 (-61.6, 74.7) | 4 |
| 40 – 49 | 20.6 (11.9, 27.7) | 24 | 34.4 (19.8, 46.1) | 40 | 68.8 (39.6, 92.3) | 80 |
| 50 – 59 | 10.4 (-1.5, 21.5) | 41 | 17.4 (-2.5, 35.8) | 69 | 34.7 (-5.0, 71.6) | 137 |
| 60 – 69 | 3.2 (-9.2, 14.9) | 30 | 5.4 (-15.4, 24.8) | 50 | 10.8 (-30.8, 49.5) | 101 |
| 70 – 79 | 4.1 (-12.4, 17.6) | 74 | 6.8 (-20.7, 29.3) | 122 | 13.5 (-41.3, 58.6) | 243 |
| Overall\|\| | 6.7 (-1.1, 13.7) | 25 | 11.2 (-1.8, 22.8) | 42 | 22.3 (-3.5, 45.5) | 84 |

GIF, Generalized Impact Fraction in percent; SI, 2.5 % to 97.5% Simulation Interval from 10,000 bootstrapped data sets.

*30% reduction in total cholesterol levels ≥ 5.18 mmol/l (200 mg/dl) to ideal levels < 5.18 mmol/l.

†50% reduction total cholesterol levels ≥ 5.18 mmol/l (200 mg/dl) to ideal levels < 5.18 mmol/l.

‡100% reduction total cholesterol levels ≥ 5.18 mmol/l (200 mg/dl) to ideal levels < 5.18 mmol/l.

§The preventable number of MI per 100,000 person-years.

||The overall GIF using the case-load weighted sum method.
