# Supplementary material for: Cardiovascular health and the modifiable burden of incident myocardial infarction: the Tromsø Study
Source: BMC Public Health. 2015 Mar 6;15:221. doi: 10.1186/s12889-015-1573-0 (PMC4355366; doi:10.1186/s12889-015-1573-0)
Supplement: Additional file 5: Table S5. — Generalized Impact Fraction of reduction in blood pressure by age and sex. The Tromsø Study 1994-2008. [file 12889_2015_1573_MOESM5_ESM.docx]

Supplemental Table 5. Generalized Impact Fraction of reduction in blood pressure by age and sex. The Tromsø Study 1994-2008.

|  | Scenario 1* | | Scenario 2† | | Scenario 3‡ | |
| --- | --- | --- | --- | --- | --- | --- |
| Baseline age, years | GIF (95% SI) | Prev, no§ | GIF (95% SI) | Prev, no§ | GIF (95% SI) | Prev, no§ |
| Men |  |  |  |  |  |  |
| 30 – 39 | 9.3 (-4.6, 21.6) | 19 | 15.5 (-7.7, 36.0) | 31 | 30.9 (-15.5, 72.0) | 62 |
| 40 – 49 | 6.8 (-3.7, 15.9) | 28 | 11.4 (-6.1, 26.5) | 48 | 22.7 (-12.2, 52.9) | 95 |
| 50 – 59 | 14.3 (5.6, 22.0) | 134 | 23.9 (9.4, 36.7) | 223 | 47.8 (18.8, 73.3) | 446 |
| 60 – 69 | 10.3 (-1.2, 20.1) | 190 | 17.1 (-2.0, 33.6) | 315 | 34.3 (-4.0, 67.1) | 632 |
| 70 – 79 | 9.4 (-6.7, 21.9) | 335 | 15.6 (-11.2, 36.4) | 556 | 31.3 (-22.5, 72.9) | 1115 |
| Overall\|\| | 10.4 (4.6, 15.3) | 80 | 17.3 (7.7, 25.5) | 133 | 34.6 (15.5, 51.1) | 265 |
| Women |  |  |  |  |  |  |
| 30 – 39 | 20.4 (-3.5, 24.8) | 3 | 34.0 (-5.9, 41.4) | 4 | 68.0 (-11.7, 82.7) | 9 |
| 40 – 49 | 19.7 (12.1, 26.3) | 23 | 32.9 (20.2, 43.8) | 38 | 65.7 (40.4, 87.6) | 76 |
| 50 – 59 | 18.2 (9.7, 25.4) | 72 | 30.3 (16.1, 42.3) | 119 | 60.6 (32.3, 84.6) | 239 |
| 60 – 69 | 9.7 (-4.4, 21.2) | 91 | 16.1 (-7.3, 35.4) | 150 | 32.2 (-14.7, 70.8) | 300 |
| 70 – 79 | 16.8 (2.8, 26.7) | 302 | 28.1 (4.6, 44.5) | 506 | 56.1 (9.2, 88.9) | 1009 |
| Overall\|\| | 15.1 (8.1, 20.7) | 57 | 25.2 (13.5, 34.5) | 95 | 50.4 (27.0, 69.0) | 189 |

GIF, Generalized Impact Fraction in percent; SI, 2.5 % to 97.5% Simulation Interval from 10,000 bootstrapped data sets.

*30% reduction in subjects with SBP ≥ 120 or DBP ≥ 80 mmHg to levels SBP < 120 mmHg and DBP < 80 mmHg.

†50% reduction in subjects with SBP ≥ 120 or DBP ≥ 80 mmHg to levels SBP < 120 mmHg and DBP < 80 mmHg.

‡100% reduction in subjects with SBP ≥ 120 or DBP ≥ 80 mmHg to levels SBP < 120 mmHg and DBP < 80 mmHg.

§The preventable number of MI per 100,000 person-years.

||The overall GIF using the case-load weighted sum method.
