# Supplementary material for: Cardiovascular health and the modifiable burden of incident myocardial infarction: the Tromsø Study
Source: BMC Public Health. 2015 Mar 6;15:221. doi: 10.1186/s12889-015-1573-0 (PMC4355366; doi:10.1186/s12889-015-1573-0)
Supplement: Additional file 6: Table S6. — Generalized Impact Fraction of reduction in smoking by age and sex. The Tromsø Study 1994-2008. [file 12889_2015_1573_MOESM6_ESM.docx]

Supplemental Table 6. Generalized Impact Fraction of reduction in smoking by age and sex. The Tromsø Study 1994-2008.

|  | Scenario 1* | | Scenario 2† | | Scenario 3‡ | |
| --- | --- | --- | --- | --- | --- | --- |
| Baseline age, years | GIF (95% SI) | Prev, no§ | GIF (95% SI) | Prev, no§ | GIF (95% SI) | Prev, no§ |
| Men |  |  |  |  |  |  |
| 30 – 39 | 16.6 (12.0, 21.1) | 33 | 27.7 (20.1, 35.1) | 56 | 55.4 (40.2, 70.2) | 111 |
| 40 – 49 | 6.7 (3.6, 9.9) | 28 | 11.2 (5.9, 16.5) | 47 | 22.4 (11.9, 33.0) | 93 |
| 50 – 59 | 8.3 (5.7, 10.9) | 78 | 13.8 (9.5, 18.2) | 129 | 27.6 (19.0, 36.3) | 258 |
| 60 – 69 | 2.3 (0.0, 4.8) | 42 | 3.9 (0.0, 8.0) | 72 | 7.8 (0.0, 16.0) | 144 |
| 70 – 79 | 1.5 (-0.8, 3.9) | 53 | 2.5 (-1.3, 6.5) | 89 | 5.0 (-2.6, 13.0) | 178 |
| Overall\|\| | 5.5 (4.3, 6.8) | 43 | 9.3 (7.2, 11.4) | 71 | 18.5 (14.3, 22.8) | 142 |
| Women |  |  |  |  |  |  |
| 30 – 39 | NA | NA | NA | NA | NA | NA |
| 40 – 49 | 23.2 (18.5, 27.5) | 27 | 38.7 (30.9, 45.9) | 45 | 77.5 (61.7, 91.7) | 90 |
| 50 – 59 | 9.6 (6.0, 13.3) | 38 | 16.0 (9.9, 22.2) | 63 | 32.0 (19.9, 44.5) | 126 |
| 60 – 69 | 4.9 (2.2, 7.6) | 46 | 8.1 (3.6, 12.7) | 76 | 16.2 (7.3, 25.4) | 151 |
| 70 – 79 | 3.7 (1.6, 6.0) | 67 | 6.1 (2.6, 10.0) | 110 | 12.3 (5.2, 20.0) | 221 |
| Overall\|\| | 7.1 (5.6, 8.7) | 27 | 11.9 (9.3, 14.5) | 44 | 23.8 (18.6, 29.1) | 88 |

GIF, Generalized Impact Fraction in percent; SI, 2.5 % to 97.5% Simulation Interval from 10,000 bootstrapped data sets.

*30% reduction in daily smokers to non-smokers.

†50% reduction in daily smokers to non-smokers.

‡100% reduction in daily smokers to non-smokers.

§The preventable number of MI per 100,000 person-years.

||The overall GIF using the case-load weighted sum method.
