# Supplementary material for: Cardiovascular health and the modifiable burden of incident myocardial infarction: the Tromsø Study
Source: BMC Public Health. 2015 Mar 6;15:221. doi: 10.1186/s12889-015-1573-0 (PMC4355366; doi:10.1186/s12889-015-1573-0)
Supplement: Additional file 7: Table S7. — Generalized Impact Fraction of a decrease in unfavorable physical activity levels by age and sex. The Tromsø Study 1994-2008. [file 12889_2015_1573_MOESM7_ESM.docx]

Supplemental Table 7. Generalized Impact Fraction of a decrease in unfavorable physical activity levels by age and sex. The Tromsø Study 1994-2008.

|  | Scenario 1* | | Scenario 2† | | Scenario 3‡ | |
| --- | --- | --- | --- | --- | --- | --- |
| Baseline age, years | GIF (95% SI) | Prev, no§ | GIF (95% SI) | Prev, no§ | GIF (95% SI) | Prev, no§ |
| Men |  |  |  |  |  |  |
| 30 – 39 | 0.9 (-5.0, 6.7) | 2 | 1.5 (-8.3, 11.1) | 3 | 3.0 (-16.7, 22.2) | 6 |
| 40 – 49 | 1.6 (-2.8, 5.9) | 7 | 2.7 (-4.7, 9.8) | 11 | 5.3 (-9.5, 19.6) | 22 |
| 50 – 59 | 2.4 (-1.4, 6.2) | 22 | 4.0 (-2.4, 10.4) | 37 | 8.0 (-4.8, 20.8) | 75 |
| 60 – 69 | 5.5 (2.0, 8.9) | 101 | 9.2 (3.3, 14.9) | 169 | 18.3 (6.6, 29.8) | 337 |
| 70 – 79 | 6.7 (2.8, 10.2) | 239 | 11.1 (4.7, 17.1) | 396 | 22.2 (9.4, 34.2) | 791 |
| Overall\|\| | 3.9 (2.0, 5.7) | 30 | 6.6 (3.4, 9.5) | 50 | 13.1 (6.8, 18.9) | 100 |
| Women |  |  |  |  |  |  |
| 30 – 39 | 20.8 (-0.6, 25.1) | 3 | 34.7 (-1.0, 41.9) | 4 | 69.5 (-1.9, 83.8) | 9 |
| 40 – 49 | 12.7 (5.3, 19.7) | 15 | 21.2 (8.9, 32.8) | 25 | 42.3 (17.8, 65.6) | 49 |
| 50 – 59 | 10.8 (5.2, 16.0) | 43 | 18.1 (8.7, 26.7) | 71 | 36.1 (17.5, 53.5) | 142 |
| 60 – 69 | 0.3 (-5.2, 5.5) | 3 | 0.5 (-8.6, 9.2) | 5 | 1.0 (-17.2, 18.4) | 9 |
| 70 – 79 | 4.5 (-0.7, 9.5) | 81 | 7.5 (-1.2, 15.8) | 135 | 14.9 (-2.3, 31.7) | 268 |
| Overall\|\| | 5.4 (2.4, 8.1) | 20 | 9.0 (4.0, 13.5) | 34 | 17.8 (8.1, 26.9) | 67 |

GIF, Generalized Impact Fraction in percent; SI, 2.5 % to 97.5% Simulation Interval from 10,000 bootstrapped data sets.

*30% decrease in subjects in the sedentary and intermediate group to the ideal physical activity group.

†50% decrease in subjects in the sedentary and intermediate group to the ideal physical activity group.

‡100% decrease in subjects in the sedentary and intermediate group to the ideal physical activity group.

§The preventable number of MI per 100,000 person-years.

||The overall GIF using the case-load weighted sum method.
