# Supplementary material for: Cardiovascular health and the modifiable burden of incident myocardial infarction: the Tromsø Study
Source: BMC Public Health. 2015 Mar 6;15:221. doi: 10.1186/s12889-015-1573-0 (PMC4355366; doi:10.1186/s12889-015-1573-0)
Supplement: Additional file 8: Table S8. — Generalized Impact Fraction of reduction in diabetes by age and sex. The Tromsø Study 1994-2008. [file 12889_2015_1573_MOESM8_ESM.docx]

Supplemental Table 8. Generalized Impact Fraction of reduction in diabetes by age and sex. The Tromsø Study 1994-2008.

|  | Scenario 1* | | Scenario 2† | | Scenario 3‡ | |
| --- | --- | --- | --- | --- | --- | --- |
| Baseline age, years | GIF (95% SI) | Prev, no§ | GIF (95% SI) | Prev, no§ | GIF (95% SI) | Prev, no§ |
| Men |  |  |  |  |  |  |
| 30 – 39 | NA | NA | NA | NA | NA | NA |
| 40 – 49 | 2.7 (0.8, 4.9) | 11 | 4.6 (1.4, 8.2) | 19 | 9.1 (2.7, 16.4) | 38 |
| 50 – 59 | 2.6 (0.8, 4.5) | 24 | 4.3 (1.4, 7.6) | 40 | 8.6 (2.7, 15.2) | 80 |
| 60 – 69 | 2.6 (0.9, 4.4) | 48 | 4.3 (1.6, 7.4) | 79 | 8.6 (3.1, 14.8) | 158 |
| 70 – 79 | 0.8 (-0.5, 2.4) | 29 | 1.4 (-0.8, 4.1) | 50 | 2.8 (-1.6, 8.1) | 100 |
| Overall\|\| | 2.2 (1.3, 3.1) | 15 | 3.6 (2.2, 5.2) | 26 | 7.2 (4.4, 10.3) | 51 |
| Women |  |  |  |  |  |  |
| 30 – 39 | NA | NA | NA | NA | NA | NA |
| 40 – 49 | 2.6 (0.3, 6.3) | 3 | 4.4 (0.5, 10.5) | 5 | 8.7 (1.0, 21.0) | 10 |
| 50 – 59 | 1.6 (-0.5, 3.9) | 6 | 2.6 (-0.8, 6.4) | 10 | 5.2 (-1.6, 12.9) | 21 |
| 60 – 69 | 3.8 (1.6, 6.0) | 35 | 6.3 (2.7, 10.1) | 59 | 12.6 (5.4, 20.1) | 118 |
| 70 – 79 | 2.7 (0.9, 4.6) | 49 | 4.5 (1.6, 7.6) | 81 | 8.9 (3.2, 15.2) | 160 |
| Overall\|\| | 2.8 (1.7, 4.0) | 10 | 4.7 (2.8, 6.6) | 17 | 9.3 (5.7, 13.2) | 35 |

GIF, Generalized Impact Fraction in percent; SI, 2.5 % to 97.5% Simulation Interval from 10,000 bootstrapped data sets.

*30% proportional reduction prevalence of diabetes.

†50% proportional reduction prevalence of diabetes.

‡100% proportional reduction prevalence of diabetes.

§The preventable number of MI per 100,000 person-years.

||The overall GIF using the case-load weighted sum method.
